# Advances in Single-Cell and Spatial Omics for Glioblastoma Precision Medicine

**DOI:** 10.1007/s11912-026-01847-7

**Published:** 2026-10-22

**Authors:** Liya Prakash, Ruman Rahman

**Affiliations:** Biodiscovery Institute, School of Medicine, University of Nottingham, University Park, Nottingham, NG7 2RD UK

**Keywords:** Glioblastoma, scRNA-seq, Spatial Transcriptomics, Multi-Omics, Tumour Microenvironment

## Abstract

**Purpose of Review:**

Glioblastoma is the most aggressive and fatal primary malignant brain tumour in adults, characterised by extensive intra-tumour heterogeneity, diffuse infiltration into surrounding brain tissue. Despite advances in surgery, radiotherapy, and chemotherapy, patient prognosis remains poor, underscoring the critical need for improved biological insight to guide precision medicine potential strategies. Although traditional bulk genomic and transcriptomic analyses have made a substantial contribution to understanding glioblastoma pathophysiology, such average molecular signals across cell populations obscures tumour microenvironment influence and clinically significant cellular states.

**Recent Findings:**

Recent advances in single-cell and spatial omics technologies have revolutionised glioblastoma research by enabling high-resolution analysis of tumour composition, cellular plasticity, and spatial organisation. These modalities have revealed that glioblastoma is composed of dynamic and plastic cellular states as opposed to fixed molecular subtypes, including stem-like and therapy-resistant populations that contribute to recurrence. The tumour architecture is preserved by spatially resolved transcriptomic and proteomic approaches, which reveal regional transcriptional programmes influenced by the tumour ecosystem. The findings provide a more resolved view of glioblastoma evolution and treatment resistance, challenging current classification systems. However, significant challenges remain in integrating these high-dimensional datasets, managing technical and computational demands, and translating these approaches into clinically scalable workflows which collectively hinder their translational adoption.

**Summary:**

This review aims to synthesise recent advances in glioblastoma single-cell and spatial omics, critically evaluating both advantages and limitations in understanding tumour heterogeneity and tumour microenvironmental interactions. Furthermore, it assesses the current challenges of glioblastoma precision medicine and evaluates the potential of these technologies to advance such approaches.

## Introduction

Glioblastomas (GBM) are highly aggressive primary malignant brain tumours, characterised by rapid proliferation and diffuse infiltration into adjacent brain parenchyma [1]. With a median survival of 14–16 months from diagnosis and a five-year overall survival (OS) rate of ~ 6.8%, the prognosis remains dismal despite multimodal standard-of-care [2]. A significant barrier to improving clinical outcomes is the extensive cellular and metabolic complexity of GBM. The tumour is characterised by pronounced intrinsic biological complexity, encompassing both intra-tumoral and inter-patient heterogeneity, a defining hallmark that drives tumour progression, recurrence, and therapeutic resistance [3]. Intra-tumoral diversity has been demonstrated through biopsies obtained from spatially distinct regions, revealing pronounced differences between tumour core and periphery [4]. This heterogeneity enables malignant cells to transition between distinct cellular states in response to various stimuli within the tumour microenvironment (TME), facilitating adaptation and survival under selective pressures [5].

A critical component of GBM heterogeneity lies in the presence of glioma stem-like populations [4]. These stem-like cells drive tumour initiation and maintenance by generating a diverse progeny of differentiated cancer subclones that form the bulk of the tumour mass and exhibit notable treatment resistance [6]. Furthermore, dynamic interactions within the TME critically influence tumour evolution and progression, highlighting the need for continued investigation into both the intrinsic cellular diversity and extrinsic microenvironmental factors that sustain GBM pathophysiology [4].

Recent advances in high-resolution single-cell technologies, particularly single-cell RNA sequencing (scRNA-seq), have transformed transcriptomic inference of GBM [7]. This approach has revealed diverse cellular subpopulations within gliomas, including proliferative, stem-like, mesenchymal-like, neuron-like, and immune-related populations, each contributing uniquely to tumour growth, invasion, and therapeutic resistance [8].

All these components interact to create a complex environment in which the tumour can rapidly proliferate and influence treatment responses. Although scRNA-seq has demonstrated substantial utility for insight into diverse cellular populations, it cannot offer information on spatial context, highlighting the need for the convergence of spatial omics with scRNA-seq [3], to enable visualisation of the cellular interactions and tissue architecture at a molecular level [9].

As sequencing technologies continue to advance, multi-omics analyses that integrate genomic, transcriptomic, and epigenomic data have thus become essential for delineating the molecular landscape of GBM towards a systems level readout, and guiding the development of more precise, personalised therapeutic strategies [10].

Here we provide a focused review of the recent advances in single-cell and spatial omics technologies applied to GBM, with a particular emphasis on their emerging role in precision medicine potential. Additionally, we examine the key challenges associated with these approaches, including technical complexity, data integration, interpretative limitations, and barriers to clinical translation. Through critical evaluation of these aspects, the review highlights both the transformative potential and current limitations of single-cell and spatial omics in refining therapeutic strategies which may ultimately improve patient outcomes.

## Single-Cell Transcriptomics: Unravelling Intra-Tumour Heterogeneity

### Introduction to scRNA Technologies

Sequencing technology on a single cell was first introduced by Tang et al. in 2009 [11], and since then, the process has been continuously improved and developed. Full-length transcriptome profiling, high-throughput capabilities, and high-sensitivity scRNA-seq have arisen from this breakthrough [12]. In GBM, these advances have proved transformative given the extent of both inter- and intratumoural heterogeneity that bulk sequencing cannot resolve. Patel et al. provided one of the earliest demonstrations of scRNA-seq in GBM by analysing 430 individual cells from five primary GBM tumours. Their findings exposed extensive intratumuoral transcriptional heterogeneity, encompassing oncogenic signalling, proliferation, immune activity, and hypoxia [13]. This was extended by Darmanis et al., who profiled cells across the tumour core and the surrounding infiltrated brain, showing that a substantial fraction of transcriptionally distinct tumour cells could be recovered from tissue beyond the radiographically defined tumour margin [14], a finding of direct relevance to characterising the infiltrative margin rather than the bulk tumour mass. Building upon this, Neftel et al. described an integrative model in which malignant GBM cells occupy four principal transcriptional states — neural-progenitor-like (NPC), oligodendrocyte-progenitor-like (OPC), astrocyte-like (AC), and mesenchymal-like (MES) — with plasticity between states shaped by underlying genetic drivers and the microenvironment [8].

#### Cell Isolation and Labelling

Individual cells are separated from a GBM tissue biopsy or resection sample using techniques such as dissociation through mechanical mincing, enzymatic digestion (commonly papain- or collagenase-based systems) and agitation to create a single cell suspension [15]. As GBM tissue is highly cellular but also admixed with necrotic and infiltrated non-disease parenchyma (particularly at the tumour infiltrative margin), the optimal dissociation protocol must be matched to the region sampled to protect genetic material, preserve viability, and prevent cell lysis.

Several methods are available for the isolation of single cells from the GBM cell suspension. The limiting dilution approach has several advantages in being cost-effective and accessible but has drawbacks in efficiency, whereby many wells may not contain a single cell (some wells are either empty or contain multiple cells) [15]. Fluorescence activated cell sorting (FACS) is also widely used in GBM studies, including for isolating tumour cells by 5-aminolevulinic acid (5-ALA) fluorescence status with high specificity, but limitations include the potential for cellular stress, leading to apoptosis and RNA damage, and requirement of a large initial sample size of cells [16–18]. Alternatively, laser capture microdissection allows single cells to be obtained directly from fixed tissue histological sections  [16].

Microfluidic technologies are also widely used to isolate and label single cells from GBM tissue samples, whereby droplet-based single cell sequencing methods such as DropSeq and 10x Genomics Chromium make use of these microfluidics systems [19]. Cells are mixed with barcoded gel beads and combined with oil to form nanolitre-sized droplets, each ideally containing one cell and one bead. Beads carry oligonucleotides with a universal adapter, a unique cell barcode, a specific molecular identifier (UMI), and a poly-T tail to capture mRNA. Following cell lysis, RNA hybridises to the bead-bound oligonucleotides [15]. The barcodes enable readings to be allocated to specific cells once the resultant cDNA is amplified and transformed into sequencing libraries. In contrast, microwell-based approaches pair single cells with magnetic beads carrying similar oligonucleotides, capturing RNA post-lysis [20]. Where fresh GBM tissue cannot be dissociated promptly, single-nucleus RNA sequencing (snRNA-seq) offers an alternative method that circumvents enzymatic dissociation, reducing dissociation-associated transcriptional stress and permitting inclusive sampling of the tumour microenvironment from frozen tissue [21, 22].

#### Library Preparation

Captured RNA undergoes reverse transcription to form first-strand cDNA, followed by amplification by either polymerase chain reaction (PCR) or in vitro transcription (IVT) [23]. The cDNA library construction consists of cDNA fragmentation, adapter ligation and selection of cDNA fragments of suitable length. This RNA-based cDNA library workflow is distinct from single-cell/single-nucleus DNA sequencing (scDNA-seq), in which whole-genome amplification of genomic DNA is used to directly call copy number variants (CNVs), single nucleotide variants (SNVs), and indels [15]. scRNA-seq itself does not amplify or sequence genomic DNA and therefore cannot directly determine DNA-level variants by this route. In GBM studies, whole-genome amplification strategies are chosen according to a given target: larger regions for inferring CNV profiles used to distinguish neoplastic from non-neoplastic nuclei (e.g., for resolving tumour-infiltrated margin tissue) or shorter sequences for detecting SNVs and indels associated with GBM driver mutations such as EGFR, PDGFRA, or TERT promoter alterations [15].

#### Sequencing

Most single cell sequencing platforms used in GBM research utilise second-generation short-read technologies, which simultaneously read many small cDNA fragments but require a multi-step library preparation and amplification process [15]. In contrast, third generation long-read technologies, such as Pacific Biosciences (PacBio) single-molecule real-time (SMRT) sequencing and Oxford Nanopore Technologies (ONT) nanopore sequencing, are becoming utilised more frequently in genomic and transcriptomic studies of GBM, though their workflows and performance characteristics differ substantially by platform and by whether DNA or RNA is being sequenced [24, 25]. For DNA, both platforms can sequence native molecules directly, without a requirement for PCR amplification. PacBio’s high-fidelity (HiFi) approach uses circular consensus sequencing, in which a single DNA molecule is read repeatedly, to generate reads typically in the range of 15–20 kb at a per-read accuracy of approximately 99.9% [25]. ONT read lengths are more variable and library-dependent: typical protocols yield reads from under a kilobase up to several tens of kilobases, while ultra-long library preparations can exceed 100 kb; raw per-read accuracy has historically been lower than PacBio HiFi, although this gap has narrowed considerably with newer flow cells, chemistries, and basecalling algorithms [24]. For RNA, the two platforms are not equivalent: PacBio’s Iso-Seq workflow requires reverse transcription of RNA to cDNA before sequencing full-length transcripts, whereas ONT uniquely offers a native/direct RNA sequencing mode that sequences RNA molecules without reverse transcription or amplification [24, 25]. Long reads, whether from DNA or RNA workflows, make it possible to analyse base alterations in repetitive parts of the genome (transposons or centromeres), where short reads cannot be uniquely mapped, and to detect nucleic acid modifications in single molecules at base resolution [26]. This approach is of growing interest in GBM for resolving structural variants and extrachromosomal DNA (ecDNA) amplifications, which are common drivers of oncogene amplification in this tumour type. It also provides insight into epigenetic heterogeneity and genetic variants relevant to GBM sub-clonal variance. However, compared to short-read technology, they offer lower per-read accuracy. Currently, hybrid techniques integrating short and long reads are emerging [15].

#### Bioinformatic Analysis

**Fig. 1:**
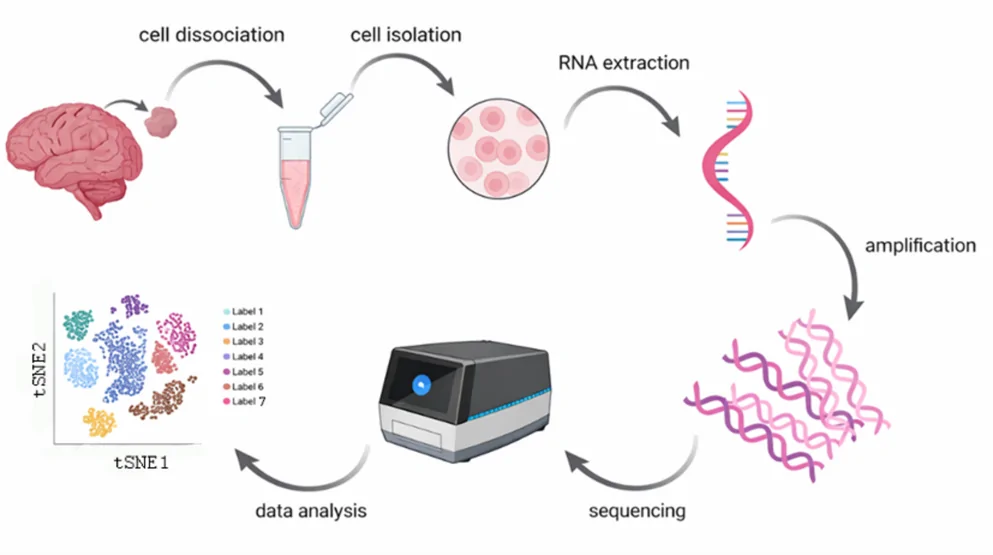
Schematic overview of the scRNA-seq workflow. Created in https://BioRender.com

Bioinformatic analysis begins with quality control of sequencing reads, including assessment of read quality, elimination of adapters and suboptimal sequences, alignment to a reference genome, and filtering of PCR duplicates [27]. Variant calling may also be attempted directly from scRNA-seq reads, but this is limited to expressed transcripts and is confounded by allelic dropout, uneven transcript coverage, and RNA-editing events that can mimic true SNVs. As such, high-confidence SNV/indel discovery still requires DNA-level sequencing. Large-scale copy number profiles can however be inferred from scRNA-seq data using tools (e.g., inferCNV, CopyKAT, HoneyBADGER) that detect shifts in average expression across contiguous genomic windows relative to a reference (typically non-malignant) cell population. These inferred CNV profiles are computational estimates of relative dosage and should not be equated with copy number alterations measured directly at the DNA level. In GBM, this step is often supplemented by inferred CNV analysis to separate malignant from non-malignant cells, an approach central to distinguishing infiltrative margin tumour cells from reactive or normal brain parenchyma [9]. One of the most significant benefits of single cell sequencing is its capacity to explore cellular heterogeneity [15]. In GBM, this is achieved by applying dimensionality reduction methods and clustering algorithms to group cells with similar transcriptional profiles, followed by classification against established malignant cell-state frameworks (such as the NPC-, OPC-, AC-, and MES-like states) and molecular subtype analysis [8, 28]. Together, these analyses can reconstruct the diverse cellular composition of GBM tissue, from the tumour core through to the infiltrative margin, and the surrounding tumour microenvironment [15] (Fig. 1).

#### Single Cell Multi-Omics

As intracellular regulatory dynamics and the translation of genomic and transcriptome heterogeneity into proteomic function are not sufficiently explored with scRNA-seq alone [15], single-cell technologies have evolved towards multimodal techniques that incorporate transcriptome data with epigenomic and proteomic layers [29].

High-resolution multi-omics profiling has been made possible by molecular barcoding and cell isolation techniques; however, tissue dissociation interferes with cell-cell interactions and spatial context [29]. To overcome these limitations, new methods combine advanced imaging technologies, such as Matrix-Assisted Laser Desorption/Ionization Mass Spectrometry Imaging (MALDI-MSI), with probe-based labelling of transcripts, proteins, lipids, and metabolites [30]. This allows for spatially resolved multi-omics mapping with enhanced sensitivity, resolution, and scalability in heterogeneous tissues.

The ability of single-cell multi-omics to identify the regulatory processes associated with genetic, epigenetic, and phenotypic variation and to resolve cellular heterogeneity, is a particular advantage. These methods make it easier to identify rare cellular states, distinguish functional subpopulations, and describe intercellular communication networks [31]. For GBM, this allows for the identification of key biomarkers to validate therapeutic strategies and improve precision treatment.

Collectively, single-cell and multi-omics technologies provide an essential foundation for precision medicine potential in GBM. However, the loss of spatial context inherent to dissociative single-cell methods highlights the importance of spatially resolved transcriptomic technologies, which are increasingly being integrated with single-cell approaches to achieve a holistic, in situ landscape of GBM architecture and cellular interactions.

### Intra-Tumoural and Inter-Tumoural Heterogeneity in GBM

Inter-patient heterogeneity in GBM is largely caused by genetic changes, including deletions or inactivating alterations in TP53, PTEN, NF1, and CDKN2A/B, as well as amplification of EGFR, PDGFRA, and CDK4/6 [32]. With advances in next-generation sequencing and computational analysis, a classification refined by Verhaak et al., reported four transcriptional subtypes: proneural, neural, classical, and mesenchymal [33]. Subsequent analyses incorporating single-cell resolution demonstrated that the neural subtype does not represent an intrinsic malignant transcriptional programme but instead arises from contamination of bulk tumour samples by non-neoplastic neural tissue. As no glioma-intrinsic gene expression cluster was found to be enriched for the neural signature once non-malignant cells were more stringently excluded, the neural subtype was subsequently removed from the classification [34, 35]. Notably, scRNA-seq has shown that GBM tumours display intratumoural plasticity where these transcriptional states frequently coexist within individual tumours and shift over time and in response to treatments. This is a caveat to considering single GBM molecular subtype classification for clinical trial stratification.

Single-nucleus RNA sequencing (snRNA-seq) is another transcriptomic technique that profiles gene expression from isolated nuclei rather than whole cells, complementing scRNA-seq [36]. Four recurrent GBM cellular states have been identified by single-cell analyses: neural progenitor cell (NPC)-like, astrocyte-like (AC-like), oligodendrocyte progenitor cell (OPC)-like, and mesenchymal-like (MES-like) [37] (summarised in Table 1 in the context of GBM transcriptomic classification schemes to date). This indicates that GBM tumour cells can adopt transcriptional programs that resemble various stages of neural development. This supports a hypothesis of lineage and cell-state plasticity by showing that stem-like characteristics in GBM are preserved throughout many lineage-associated programs, rather than representing a single fixed cell type. Notably, intra-tumour heterogeneity is highlighted by the observation that different states often coexist within individual tumours, with their relative abundance associated with specific recurrent copy-number and mutational alterations, including amplification of CDK4 (NPC-like), PDGFRA (OPC-like), and EGFR (AC-like), and NF1 mutation/loss (MES-like) [8]. These associations were derived from correlating genomic and transcriptomic data across large patient cohorts rather than from direct functional perturbation, and therefore indicate a statistical link between genotype and state abundance rather than direct evidence that these alterations causally select for a given state.

**Table 1:** Principal transcriptomic classification schemes in glioblastoma (GBM). Summary of the major bulk, single-cell/single-nucleus, and spatial transcriptomic classification frameworks applied to GBM, their associated nomenclature, the assay platforms on which each was derived, and the biological interpretation each scheme was designed to capture

Classification Scheme	Terminology / Categories	Assay Platform(s)	Intended Biological Interpretation
TCGA / Verhaak molecular subtypes [33]	Proneural Neural Classical Mesenchymal	Bulk microarray / bulk RNA expression profiling (~ 200 tumours, TCGA cohort)	First systematic, genome-wide transcriptomic classification of GBM, cross-referenced to DNA copy-number and mutation data. Intended to assign each tumour a single dominant subtype linked to characteristic genomic lesions (PDGFRA/IDH1 – Proneural; EGFR – Classical; NF1 – Mesenchymal), with the goal of stratifying patients for prognosis and subtype-directed therapy.
Wang / TCGA-refined subtypes [38]	Proneural Classical Mesenchymal (Neural subtype subsequently omitted)	Bulk RNA-seq with computational purification for tumour-cell content	Refined the Verhaak scheme after recognising that the “Neural” subtype largely reflected contaminating normal brain tissue rather than tumour-intrinsic biology. Reframed the three remaining subtypes as tumour-intrinsic states that can shift over time, particularly a Proneural-to-Mesenchymal transition at recurrence.
Patel et al. single-cell heterogeneity [13]	Intratumoral coexistence of multiple bulk-defined subtype signatures within one tumour	scRNA-seq (full-length, Smart-seq)	Demonstrated that individual tumour cells within a single GBM express transcriptional programmes spanning multiple Verhaak subtypes simultaneously. Reframed GBM subtyping as a question of intratumoral composition.
Neftel et al. cellular states model [8]	Four malignant cell states: NPC-like, OPC-like, AC-like, MES-like (with hybrid/transitional states)	scRNA-seq (Smart-seq2 and 10x Genomics, multiple cohorts)	Reconceptualised GBM subtyping around neurodevelopmental lineage programmes. Each tumour is modelled as a plastic ecosystem in which malignant cells occupy and interconvert between four states resembling neural/oligodendrocyte progenitor and astrocytic/mesenchymal lineages, with state proportions influenced by genetic drivers (e.g. CDK4, PDGFRA, EGFR, NF1) and the microenvironment.
Garofano et al. pathway-based classification [39]	Proliferative/progenitor, Neuronal, Mitochondrial, Glycolytic/plurimetabolic	Bulk RNA-seq and scRNA-seq (pathway-activity / gene-set based scoring)	Moves from gene-signature clustering to pathway-activity scoring, aiming to capture functional and metabolic state rather than transcript identity alone. Intended to reveal therapeutically actionable vulnerabilities (e.g. oxidative-phosphorylation dependence in the Mitochondrial subtype) that are prognostically and mechanistically distinct from the classical TCGA groups.
Ivy Glioblastoma Atlas Project [40]	Anatomic transcriptional structures: leading edge, infiltrating tumour, cellular tumour, perinecrotic/pseudopalisading zone, microvascular proliferation	Spatial transcriptomics (anatomically anchored laser-capture microdissection RNA-seq / ISH reference)	Maps transcriptional programmes onto histologically defined anatomic niches within and around the tumour rather than onto a single tumour-wide subtype. Directly relevant to infiltrative-margin biology, where hypoxia-, invasion- and mesenchymal-associated programmes are spatially concentrated at the tumour edge and perinecrotic regions.
Richards et al. developmental hierarchy model [41]	Conserved stem-like, progenitor-like, differentiated-like hierarchy, spatially organised around a hypoxic/perivascular niche axis	scRNA-seq and snRNA-seq (multi-region sampling)	Extends single-cell state models across the glioma spectrum by organising malignant cells along a conserved developmental hierarchy anchored to spatial niches (e.g. hypoxic vs. perivascular). Intended to unify cellular-state and spatial/microenvironmental classification within one interpretive framework.

These schemes are complementary rather than mutually exclusive. Bulk-based subtypes (Verhaak; Wang) describe the dominant transcriptional programme averaged across a tumour sample; single-cell/single-nucleus schemes (Patel; Neftel; Richards) resolve intratumoral heterogeneity and cell-state plasticity underlying those bulk calls; pathway-based classification (Garofano) reframes subtypes around functional/metabolic activity; and spatial transcriptomics (Ivy GAP; Richards) localises these programmes to anatomic and microenvironmental niches, including the infiltrative margin

Furthermore, using scRNA-seq, Hazra et al. identified glioma stem-like population-associated long non-coding RNAs (lncRNAs) which are defined as transcripts greater than 200nt that do not encode proteins [42]. As GBM development parallels normal human neurodevelopment, lncRNAs may play a role in driving GBM heterogeneity and resistance to therapy [43]. Glioma stem-like population -enriched lncRNAs such as GIHCG regulate proliferation, migration, and stemness, supporting a model of lncRNA-driven glioma stem-like population plasticity and transcriptional heterogeneity that contributes to GBM adaptability and therapeutic resistance [42]. However, no clinically approved therapies specifically target glioma stem-like populations, highlighting a clinical gap. Expanded identification and functional characterisation of lncRNAs is therefore required to establish effective GSC-specific targets.

### Cellular State Transitions and Therapy Resistance

#### Tumour Intrinsic Resistance

Single-cell transcriptomic studies have revealed that GBM resistance is closely linked to tumour-intrinsic transcriptional plasticity rather than fixed cellular identities. Neftel et al. identified four primary malignant cell states that are highly interconvertible and mirror neural lineage programs: NPC-like, OPC-like, AC-like, and MES-like [8]. The ability of GBM cells to flexibly switch between phenotypes is indicated by this underlying heterogeneity, whereby GBM cells occupy a continuum of interconvertible developmental and reactive states which can be seen on a ‘reactive-developmental programs’ axis, which serves as a substrate for adaptive resistance under therapeutic stress [44].

Subsequent scRNA and snRNA-seq studies have demonstrated that standard therapy confers strong selective pressure on these transcriptional states, most notably driving a PN-to-MES shift at recurrence [34]. Specifically, paired analyses of primary and recurrent GBM samples have shown a significant increase in the proportion and proliferative activity of MES cells at recurrence, indicating mesenchymal transition as a hallmark of therapy resistance [34]. Additionally, gene expression programs associated with the return of earlier quiescent MES cells into the cell cycle were identified, and inhibiting these programs reduced GBM cell viability, highlighting their functional significance [45].

#### TME Driven Resistance

In parallel with tumour-intrinsic plasticity, the TME plays a critical role in reinforcing resistant cellular states. Single-cell analyses have shown that paracrine and autocrine signalling between neoplastic cells, neuroglial populations, and immune cells, supports the expansion and survival of MES-like tumour cells, particularly at recurrence [34]. MES-like tumour cells are frequently enriched at tumour margins, where they promote invasion, migration, and angiogenesis [3]. In response to external stresses such as therapy or hypoxia, stem-like cancer cells can further differentiate into proliferative or MES-like states, demonstrating how TME signals actively influence tumour adaptability, including post-treatment [46].

Resistance is also facilitated by adaptive remodelling of the immune microenvironment. The predominant immunosuppressive tumour-associated macrophages (TAMS) in GBM are monocyte-derived macrophages, which proliferate during recurrence. De Leo et al. demonstrated that metabolic reprogramming within these TAMs, such as PERK-mediated glycolysis leading to histone lactylation, enhances their immunosuppressive function, further supporting tumour persistence [47].

#### Phenotypic Plasticity

Therapy-induced resistance in GBM is increasingly understood as a consequence of dynamic TME interactions rather than manifesting from tumour-intrinsic genetic changes alone. Transcriptomic studies initially suggested a reduction in blood derived monocytes following treatment; however, these bulk-level observations mask important state-dependent and spatially restricted immune changes [45]. More recent single-cell analyses challenge earlier bulk-derived conclusions by demonstrating an expansion of monocyte-derived macrophages in recurrent tumours, alongside increased CD8⁺ T-cell infiltration in hypermutated GBMs [48]. This emphasises how investigating adaptive resistance mechanisms within the TME requires single-cell resolution.

Importantly, growing research indicates that TME populations are not passive bystanders but actively undergo phenotypic adaptation that reinforces therapeutic resistance. Tumour survival is enhanced by radiation-induced macrophage states, which demonstrate how immune cells can be reprogrammed into phenotypes that encourage resistance [45]. This reversibility implies that, if interventions are timely and state-specific, some resistance mechanisms may be therapeutically targeted efficaciously.

Tumour cell plasticity also contributes to resistance to molecular targeted therapies. Receptor tyrosine kinase inhibition (RTKI) often causes adaptive signalling rewiring and entry into drug-tolerant persister states rather than selecting for stable resistant clones [49]. For example, dasatinib-induced PDGFRA inhibition causes reversible transitions into quiescent Notch- and KDM6-dependent states, together with H3K27 histone mark epigenetic remodelling and neurodevelopmental program reactivation [45]. Importantly, these persister states can be present in tumours that are treatment-naive, indicating that therapeutic failure might not be due to uniform therapy-induced reprogramming, but rather due to the variable growth of latent resistant states.

This supports a model in which resistance emerges from preexisting heterogeneity and therapy-induced adaptation. Two non-exclusive processes appear to operate in parallel: (1) selection and expansion of preexisting resistant cellular states, and (2) adaptive reprogramming of tumour and microenvironmental cells into drug-tolerant phenotypes [34, 47, 50]. From a precision-medicine perspective, this plasticity implies that static molecular stratification is insufficient; instead, effective therapy will require dynamic monitoring and co-targeting of both tumour cell states and the adaptive microenvironmental programs that sustain resistance.

### Future Outlook: Towards Translational Single-Cell Profiling

The goal of GBM precision medicine is to identify molecular characteristics that are diagnostic, prognostic and therapeutic [51]. Wang et al. demonstrated that infiltrating GBM cells undergo marked molecular alterations by collecting paired tissues from the tumour core (TC) and peritumoural brain (PTB) for integrated snRNA-seq and snATAC-seq analyses [52]. The tumour molecular identity varied by tumour location (core vs. periphery) and this spatial heterogeneity contributes substantially to the tumour’s treatment resistance. At the transcriptome level, tumour cells invading PTB from TC resembles oligodendrocyte progenitor cells in comparison to astrocytes. Dual-omics data suggest that regional differences are driven by distinct transcriptional regulators: activator protein (AP-1) activity diminishes from core to periphery, whereas BACH1 increases. In mouse models, simultaneously targeting AP-1 and BACH1 reduces tumour growth more effectively and extends survival compared with single target therapy [52].

Another avenue for precision medicine potential GBM is tumour-intrinsic profiling combined with microenvironmental and ecosystem-level profiling. Myeloid cells and other immune cells within GBM contribute to immunosuppression, T-cell dysfunction, and therapy resistance [53]. Therefore, targeting those non-tumour components (e.g. TAMs/ microglia, suppressive myeloid subsets, dysfunctional immune niches) may offer a novel therapeutic approach that could improve treatment efficacy. However, a drawback of scRNA-seq is technical noise arising from amplification bias and dropout events which obscure functionally important cells [51].

Nevertheless, the use of scRNA-seq has contributed to understanding GBM relapse pathways. For instance, relapsed GBM in comparison to primary tumours exhibit mutations in the RAS/GEF GTP-dependent signalling pathway, which sheds light on the molecular alterations driving treatment resistance [54]. The potential of using scRNA-seq to create prognostic models in GBM has also been investigated in several studies with potential translation to improve clinical outcomes. For instance, Lai et al. integrated a scRNA-seq and bulk RNA‐seq dataset to build a prognostic model for predicting survival in GBM [55]. This study developed a ‘five-gene-based risk score prognostic model’ based on 43 differentially expressed genes that were linked to overall survival in both datasets. However, such a model associated with overall survival is not necessarily predictive of a potential treatment benefit. Furthermore, the model did not incorporate broader clinical patient data, which, if incorporated, may enhance its clinical relevance and accuracy [55].

As liquid biopsies gain prominence in modern molecular diagnostics, scRNA-seq shows considerable promise for early cancer diagnosis [51]. However, as a relatively nascent field, standardised frameworks for quality control, data processing, and interpretation have yet to be fully established, limiting reproducibility and clinical translation. Therefore, to obtain more reliable insights into CSF roles in GBM, larger studies with better defined cohorts and standardised methods are needed [56].

Ultimately, the translational goal is to bring rapid, sequencing-based molecular profiling into real time surgical and therapeutic decision-making in GBM care [53]. Proof-of-concept for this is already emerging in the intraoperative setting, where nanopore-based DNA methylation classifiers can deliver a provisional tumour classification within approximately two hours of tissue sampling. Subsequently, comprehensive SNV, CNV, and structural variant profiling completed within 24 h and shown to inform surgical strategy, achieving up to 90% concordance with final integrated diagnosis in prospective cohorts [57, 58]. These intraoperative workflows currently rely on rapid DNA-methylation and mutational profiling rather than scRNA-seq, where longer library-preparation and analysis timelines remain a barrier to real-time use. Nevertheless, such approaches demonstrate the clinical feasibility of rapid molecular diagnostics in the neurosurgical workflow and provide a template that single-cell transcriptomic approaches may build towards, as turnaround times improve. Achieving broader integration will require a clinical framework that incorporates multi-omics data into routine workflows.

## Spatial Transcriptomics: Mapping the Tumour Microenvironment

### Overview of Spatial Omics Technologies

Given that GBM progression and therapeutic resistance are driven by spatially defined cellular niches, approaches that preserve spatial context are essential for precision medicine applications, provided that assay turnaround in a timely manner, treatment choice, and patient outcome are ultimately demonstrated in the clinic. In the context of GBM’s marked heterogeneity, cellular plasticity, and complex TME architecture, the development of modern spatial transcriptomic technologies enables the systematic mapping of cell types and their spatial relationships, facilitating the identification of distinct TME cellular neighbourhoods [59]. These insights are key for refining tumour classification, elucidating niche-specific interactions, and importantly, improving patient outcomes [59]. Spatial omics technologies, including spatial transcriptomics, proteomics and epigenomics, encompass techniques that allow the simultaneous measurement of numerous molecules in intact tissue [9]. These tools have already yielded many insights into the GBM TME, despite difficulties with data complexity and integration [60]. It is important to note that GBM specimen-preparation choice materially affects spatial transcriptomic data quality. Fresh-frozen (FF) tissue best preserves RNA integrity (RIN ≥ 7) and is required for polyA-capture platforms but demands intraoperative freezing and cold-chain handling. Formalin-fixed paraffin-embedded (FFPE) tissue offers superior morphology and archival stability; however, formalin crosslinking fragments RNA, limiting FFPE-compatible platforms to targeted probe-based chemistries. Paired comparisons show FFPE-derived expression data correlate well with FF once degraded samples are excluded, retaining the ability to support subtype classification, albeit at reduced confidence [61]. Moreover, section thickness, slide adhesion, and processing interval further influence capture efficiency and alignment in both preparation types.

These variables thus crucially shape downstream interpretation: FF tissue supports deeper, less biased cell-type deconvolution and more reliable niche delineation, whereas FFPE/degraded material remains usable for subtype- or niche-level classification but with reduced sensitivity. Large-scale spatial characterisation of GBM architecture, including invasive-margin niches, has therefore relied predominantly on FF material [62], and specimen-preparation strategy should be selected according to the transcriptomic resolution a given biological question requires.

#### Spatial Transcriptomics

Spatial transcriptomics technologies enable the localisation and quantification of gene expression by measuring mRNA transcripts within intact cells and tissues, preserving their native spatial architecture [9]. These technologies can be broadly classified into two principal categories: imaging-based and next-generation sequencing (NGS) based approaches [9]. These methodologies differ fundamentally in how spatial information and transcript abundance are captured. Imaging-based approaches detect transcripts directly within tissue sections using fluorescence in situ hybridization (FISH) or direct in situ sequencing (ISS), typically through iterative imaging and combinatorial barcoding methods, enabling transcript detection at single-molecule resolution [60]. In contrast, NGS based approaches employs spatially indexed, barcoded surfaces to capture RNA from defined regions of tissue sections, with transcript identities subsequently determined through NGS.

##### Class I: Imaging-Based Spatial Transcriptomics

These methods rely on fluorescently labelled probes that hybridise to target RNA molecules in situ, allowing gene expression to be visualised at the single-cell or subcellular level. Sequential rounds of probe hybridisation, imaging, and signal decoding, have significantly increased transcript throughput, with modern platforms capable of profiling over 20,000 genes within a single tissue segment [63]. In contrast, ISS-based approaches determine transcript identity through sequencing reactions performed directly within the tissue, allowing spatially resolved transcriptomic profiling while also enabling the detection of sequence variants, including SNVs [64]. ISS methods can be either targeted, relying on predefined gene panels, or untargeted, offering larger transcriptome coverage but lower detection performance due to difficulties such as molecular crowding [65].

Current ISH technologies include:

i) Xenium (10× Genomics): This advanced in situ transcriptomics method analyses thousands of RNA targets in FFPE (formalin-fixed paraffin-embedded) and FF (formalin-fixed) tissues. Xenium is especially useful for examining tissues with spatially variable gene expression, such as TMEs due to its high-resolution data output [66].
ii) Multiplexed Error-Robust Fluorescence In Situ Hybridization (MERFISH): This RNA imaging technology enhances single-molecule FISH by incorporating combinatorial labelling, sequential imaging, and error-robust barcoding. It allows for spatially resolved, multiplexed gene expression profiling at a 0.1 μm single-cell resolution which reduces optical crowding and enables accurate error detection and repair [60].
iii) Sequential Fluorescence In Situ Hybridization (SeqFISH+): SeqFISH+ enhances seqFISH by combining sequential and imaging using a conventional confocal microscope, making it possible to multiplex thousands of genes in a single cell [67]. SeqFISH+ effectively identifies transcripts using a combinatorial FISH technique similar to MERFISH with high spatial resolution [68].
iv) CosMx Spatial Molecular Imager (SMI): Through several cycles of nucleic acid hybridisation of fluorescent molecular barcodes, this technique uses high-resolution imaging to record the expression of thousands of RNA targets on a single slide [60].

Current ISS technologies include:

Targeted ISS: This technique focusses on preselected genes, achieves high detection efficiency, and can analyse SNVs.

i) Spatially resolved Transcript Amplicon Readout mapping (STARMAP): This approach effectively analyses whole tissue samples in 3D while keeping cell orientation of the cells for 100–150 μm thick sections  [60]. STARmap PLUS improves spatial resolution and detection accuracy with a 95 × 95 × 350 nm voxel size, surpassing STARmap’s maximum of 1024 genes [69]. This approach improves optical transparency while reducing background noise in tissues [60].

Untargeted ISS:

i) Barcode in situ targeted Sequencing (BaristaSeq) - Compared to traditional gap-fill padlock techniques, this enhanced implementation of gap padlock probe-based ISS results in a five-fold rise in amplification efficiency in cultured cell systems [64].
ii) Fluorescent In Situ RNA Sequencing (FISSEQ): This method allows for the sequencing and localisation of thousands of RNA species. FISSEQ offers analyses on early detection, the influence of gene alterations on metastasis, responsiveness to selected therapy and the identification of novel therapeutic targets [60].

Collectively, imaging-based spatial transcriptomic platforms offer subcellular spatial resolution and high target specificity, and independent benchmarking has shown substantial cross-platform concordance in gene expression measurements when platforms such as CosMx, MERFISH, and Xenium are compared using shared genes on matched tumour samples. However, reproducibility is not an intrinsic platform property, which is determined by assay design, operator and batch variation, segmentation accuracy, and the downstream analysis pipeline. Cross-platform correlation itself varies by comparison pair and tissue context rather than being uniform across platforms. Furthermore, segmentation errors can cause molecular admixture between adjacent cells that confounds differential expression and interaction analyses. Systematic non-biological variation arising from operators, reagent batches, and equipment can further obscure true biological signals. These platforms are nonetheless particularly well suited to studying fine-scale cellular organisation, cell-cell interactions, and the TME, provided such sources of technical variability are accounted for in study design and analysis [70, 71].

##### Class II: Sequencing-Based Spatial Transcriptomics

In contrast, sequencing-based ST uses NGS to provide largely unbiased, whole-transcriptome profiling while retaining spatial information [59]. However, these techniques typically compromise spatial resolution since acquired transcripts are assigned to distinct spatial capture regions potentially harbouring numerous cells.

Sequencing-based methods localise gene expression by trapping RNA molecules onto spatially barcoded surfaces, such as microarrays or bead-based substrates [63]. Spatial barcodes are added to cDNA during reverse transcription, allowing sequenced transcripts to be traced back to their original tissue sites.

10× Genomics Visium is the most widely used sequencing-based technology. It allows for whole-transcriptome profiling over extensive tissue areas utilising spatially barcoded reverse transcription primers printed on glass slides and is suitable with both FF and FFPE samples [63]. Visium does not achieve single-cell resolution due to its spot size; however, advantages include its robust transcript capture, scalability, and ease of integration into standard molecular workflows, making it ideal for translational studies.

Slide-seq and its enhanced version, Slide-seqV2, use densely packed, randomly barcoded beads that yield greater spatial resolution than Visium [72]. Slide-seqV2 achieves near-cellular resolution and significantly enhances RNA capture efficiency, allowing for the identification of cellular subpopulations and transcriptional programs similar to those discovered by scRNA-seq [73]. However, these procedures are more technically demanding and incompatible with historical FFPE materials.

Stereo-seq is a further improvement that uses DNA nanoball technology to attain nanoscale spatial resolution across extremely broad fields of vision [72]. It combines ultra-high spatial density with whole-transcriptome profiling which allows for precise mapping of tissue architecture and development. However, its current restriction to fresh frozen tissue and reliance on exclusive sequencing tools may limit its wider clinical application.

Beyond array-based techniques, the NanoString GeoMx Digital Spatial Profiler uses a region-of-interest (ROI)-based paradigm that combines spatially resolved transcriptome profiling with multiplexed protein analysis [72]. Instead of collecting complete tissue sections evenly, GeoMx allows for targeted profiling of microscopically specified locations, which provides flexibility, cost effectiveness, and compatibility with FFPE samples [74]. However, the reliance on ROI selection and the absence of actual single-cell resolution, limit fine-scale spatial analysis.

### Spatial Architecture of GBM

**Fig. 2:**
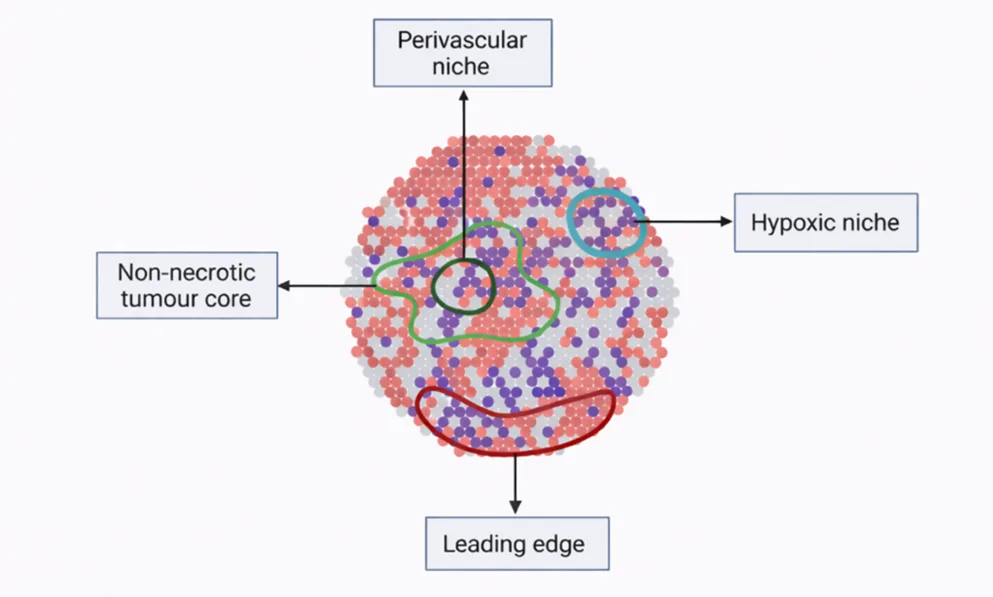
Spatial omics-based model of the GBM TME. Intra-tumour ecosystem depicting GBM as a heterogeneous population of individual cells (circles) and comprised of four spatially and functionally distinct niches: leading edge, non-necrotic tumour core, perivascular niche, and hypoxic niche. Coloured outlines and arrows demarcate the non-necrotic tumour core (green outline), the densely cellular central tumour mass excluding areas of necrosis; the perivascular niche (black circle), a peri-endothelial region associated with vascular co-option and maintenance of a stem-like tumour cell phenotype; the hypoxic niche (teal circle), a region of low oxygen tension typically found distal to functional vasculature; and the leading edge (dark red outline), the infiltrative margin at the tumour periphery where malignant cells invade adjacent non-neoplastic brain parenchyma. Created in https://BioRender.com

Many spatial transcriptomic and multi-omic studies have shown that GBM is organised into distinct but connected spatial niches, within which tumour cell states, immunological components, and metabolic programmes follow spatial patterns [75]. Although early transcriptomic initiatives, such as the Ivy Glioblastoma Atlas Project, offered valuable insights into regional gene expression, they were constrained by tissue separation and small sample sizes [75]. Since then, spatial transcriptomics has enabled more reliable mapping of cancer cell states within their natural microenvironment, particularly when paired with matched snRNA-seq from the same tissue, revealing consistent organisational principles across patients (Fig. 2).

#### Tumour Core and Perivascular Niches

A consistent finding across studies is the spatial segregation of canonical GBM tumour cell states. OPC-like tumour cells are frequently enriched within non-necrotic tumour cores and perivascular regions, whereas radial glial stem-like and neural progenitor-like states are preferentially localised to neuron-rich invasive niches in the tumour’s periphery [76]. This organisation appears conserved across GBM, implying that tumour cell identity is strongly influenced by spatial context rather than representing fixed intrinsic states. The selective localisation of OPC-like tumour cells near blood vessels is particularly significant as non-malignant OPCs rely heavily on vascular-derived oxygen, with hypoxia known to induce premature differentiation [77].

Perivascular niches serve as hubs of tumour–vascular–immune interaction. They are also enriched for diverse immune populations, including macrophages, T cells, and B cells [9]. While consensus exists regarding the co-localisation of endothelial and immune cells, discrepancies remain concerning the dominant tumour cell state adjacent to vasculature, most likely due to disparities in spatial resolution, sensitivity, and cohort size across platforms [78]. Macrophages in perivascular niches exhibit phagocytic and antigen-presenting characteristics and may operate as physical or signalling barriers to T-cell infiltration into the tumour core. Spatial and single-cell transcriptomic analyses have identified candidate ligand–receptor pairs (e.g., ANXA1–FPR1/3) computationally inferred to mediate tumour-myeloid crosstalk, with predicted downstream involvement of MAPK/ERK signalling as a possible modulator of immune suppression and treatment response. As these interactions were derived from co-expression-based inference rather than functional assays, they should be regarded as candidate signalling networks requiring experimental validation [9, 79].

#### Hypoxic and Perinecrotic Niches

Hypoxic and perinecrotic niches are another distinguishing feature of GBM’s architecture. Spatial transcriptome and metabolomic analysis have revealed that these regions harbour more MES-like tumour cells and are associated with profound metabolic reprogramming, including shifts between glycolysis and the pentose phosphate pathway [77], potentially driving cancer progression under treatment stress [9, 80]. Furthermore, hypoxic and perinecrotic zones exhibit show enrichment of macrophages and activation of immunosuppressive signalling pathways, including IL-10–associated programmes. Spatial transcriptomic and pathway-enrichment analyses suggest that macrophages in necrotic regions may be functionally distinct, with transcriptional signatures indicative of candidate immunosuppressive and tumour-supportive programmes, including cytokine-associated, lipid-handling, creatine-related, and purinergic (adenosine) pathways. These represent putative, transcriptomically inferred mechanisms that have not yet been functionally confirmed in GBM and would benefit from validation using cytokine/metabolite assays or ex vivo immune-suppression co-culture assays [77].

#### Invasive Edge and Neuron-Associated Niches

Spatial profiling has identified a discrete neuron-associated niche near the invasive tumour edge, which is enriched for stem-like tumour cells with invasive behaviour [75]. Radial glial-like tumour cells are preferentially concentrated in these areas, which are also the most neuron-rich within the tumour. Niche-specific transcriptional programs within the invasive margin suggest that local microenvironmental stimuli actively shape tumour behaviour. Functional studies have discovered drivers of invasion, such as the secreted kinase FAM20C, showing spatially constrained vulnerabilities [75]. Recent discoveries of direct neuron-glioma synaptic connections emphasise the biological significance of this niche. Tumour cells, particularly those in NPC-like or OPC-like states, form bona fide AMPA receptor-mediated synapses with neurons and receive paracrine growth signals via ADAM10-cleaved neuroligin-3 and BDNF. This has been demonstrated functionally using electrophysiological recordings, in vivo optogenetic stimulation, and pharmacological or genetic blockade of electrochemical signalling [81–83]. These validated interactions promote tumour proliferation, invasion, and microtubule branching, while bidirectional signalling increases neuronal excitability, sustaining a pro-tumorigenic feedback loop. Consistent with this circuit-level integration, spatial transcriptomic profiling shows enrichment of synaptic- and Notch-signalling gene programmes within the infiltrated tumour margin [84, 85]. Spatial transcriptomic investigations thereby collectively suggest a continuum of neuronal impact across tumour space, rather than firmly defined borders.

#### Current Limitations

Although spatial transcriptomics has yielded important GBM insights, interpretation may be impacted by bias towards densely sampled tumour areas, as this approach may underrepresent sparsely populated but biologically important regions such as infiltrative tumour margins or transitional zones between niches [86]. Other limitations include reliance on ROI selection, and technical limitations on spatial resolution [87]. Furthermore, most studies analyse relatively small tissue sections, which may not capture the full three-dimensional complexity of GBM architecture.

Ultimately, spatial transcriptomic and multi-omic studies align on a framework in which GBM is divided into four interconnected niches: the non-necrotic tumour core, hypoxic and perinecrotic mesenchymal regions, perivascular immune-rich zones, and the neuron-associated invasive edge [9]. These niches are linked by gradients in oxygenation, vascular proximity, metabolic stress, and cellular composition, allowing for dynamic transitions between tumour cell states. Ligand–receptor interactions and niche-specific mechanisms discussed throughout this review are, unless otherwise stated, computationally inferred from co-localisation and co-expression of transcripts rather than confirmed through receptor activation assays, direct contact imaging, or perturbation studies. Such analyses should therefore be interpreted as generating candidate signalling networks and hypotheses for functional testing, rather than as evidence of established mechanisms.

## Multi-Omics Integration: Bridging Genomics, Epigenomics, and Proteomics

**Fig. 3:**
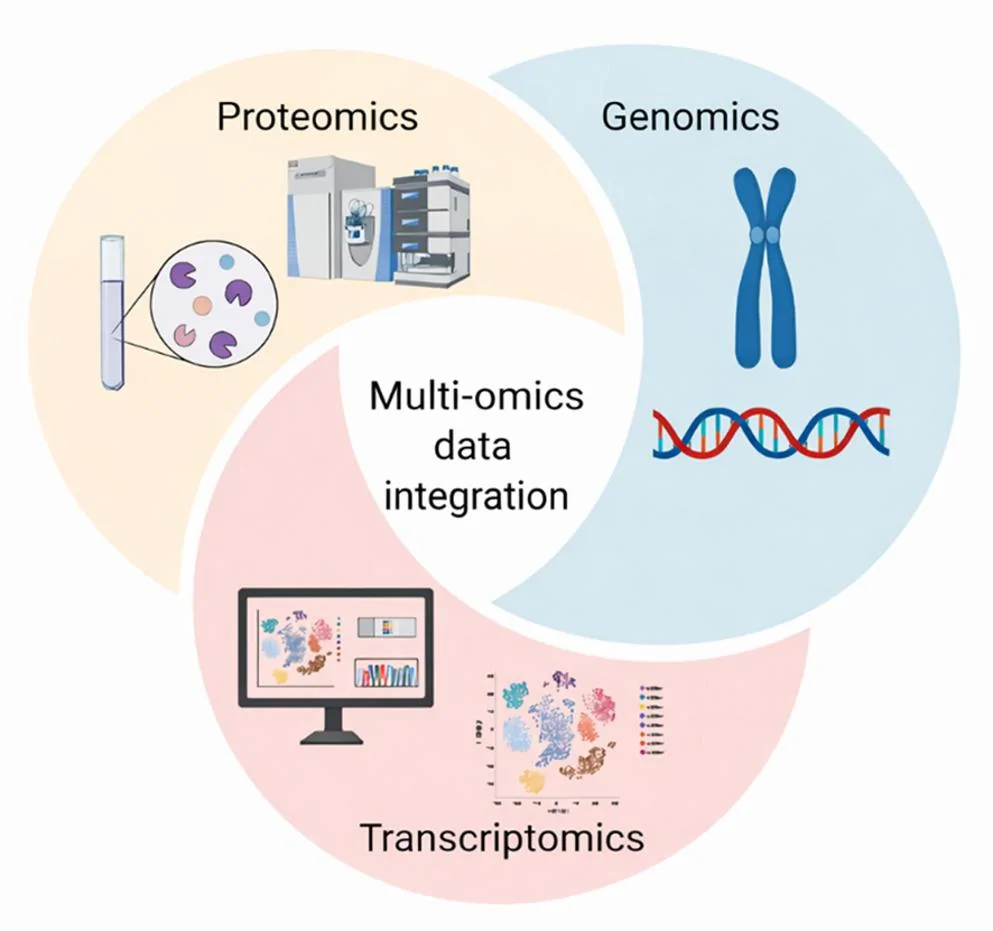
Schematic overview of multi-omics data integration. Proteomics, genomics, and transcriptomics are integrated to provide a comprehensive, systems-level view of GBM that cannot be captured by single-modality analyses alone. Created in https://BioRender.com

There is growing evidence that GBM tumour behaviour is not caused by a single molecular layer but rather by the coordinated interaction of genetic changes, epigenomic regulation, and downstream protein signalling networks [88]. While proteomic signalling networks govern biological processes underlying invasion, resistance, and recurrence, epigenomic mechanisms mould cellular identity and facilitate state changes. Therefore, an integrative, multi-omics approach that links genetic changes to regulatory control and functional output is necessary to comprehend GBM in a systems biology manner [89] (Fig. 3).

### Genomic and Epigenomic Landscapes of GBM

#### Genomic Architecture

GBM classification has been shifted by advances in genomic profiling, refining its biological understanding. GBM was redefined as an IDH-wildtype diffuse glioma with distinctive genetic features such as TERT promoter mutations, EGFR amplification, and chromosome 7 gain with chromosome 10 loss in the 2021 WHO Central Nervous System classification (WHO CNS5), which formally incorporated molecular changes into diagnostic criteria [90]. However, intratumoural heterogeneity, phenotypic plasticity, and therapeutic resistance are not explained by static genetic alterations alone, which has prompted further research into epigenomic and functional signalling layers.

#### Epigenomic Regulation

The formal integration of epigenomic mechanisms into diagnostic frameworks reflects their clinical significance with the WHO recommending DNA methylation profiling as a routine method for classifying tumours of the central nervous system.

Genome-wide methylation profiling has shown that GBM tumours with comparable transcriptional profiles can be further classified into physiologically and clinically different groups based on methylation patterns. G-CIMP is a key example, which is highly linked to IDH mutation, younger age at diagnosis, and better survival [90]. Patients with substantial hypermethylation within the PN subtype perform significantly better than their non-G-CIMP counterparts. These results emphasise DNA methylation as a regulatory layer that, rather than replicating genomic and transcriptome categories, refines them. Additionally, a predilection for MES states at recurrence has been shown through the DNA methylation subclass transition between primary and recurrent GBM, consistent with transcriptomic data [88].

IDH mutations lead to the accumulation of the oncometabolite 2-hydroxyglutarate (2-HG) which competitively inhibits α-ketoglutarate–dependent dioxygenases, including TET DNA demethylases and Jumonji-domain histone demethylases, resulting in widespread DNA hypermethylation and accumulation of repressive histone marks [88]. This pathway underpins the G-CIMP phenotype and directly connects metabolic imbalance to epigenetic reprogramming. Beyond chromatin effects, 2-HG modulates DNA repair capability, redox balance, and immune evasion, thereby integrating epigenetic and microenvironmental regulation [91].

### Proteomics and Proteogenomics

Proteogenomic integration, combining proteomics with genomic and transcriptomic data, has emerged as a critical strategy to integrate neoplastic changes with functional signalling states [92]. This strategy is demonstrated by large-scale initiatives like those carried out by the Clinical Proteomic Tumour Analysis Consortium (CPTAC), which uses coordinated multi-omic profiling to identify protein-level changes not predicted by RNA expression alone in clinically annotated GBM cohorts.

#### Proteomic Signatures of GBM Subtypes

Three main GBM proteomic categories have been discovered via proteome-based stratification: neural-like, proliferative, and immune [92]. Crucially, these proteomic states indicate functional variability across histologically comparable tumours and extend beyond traditional WHO classifications.

Proteomic studies by Van Meir et al. have further shown that subtype transitions, such as the PN-to-MES shift associated with poor prognosis, are accompanied by dynamic changes in protein expression [93]. Philips et al. found that MES tumours exhibit elevated expression of invasion-associated proteins such as YKL40, whereas Eckerdt et al. demonstrated that PN tumours show enhanced PI3K/AKT pathway activity linked to p110α signalling [94, 95]. These findings suggest that proteomic alterations may be early markers of tumour growth and phenotypic shift, possibly observable through long-term patient monitoring.

#### Phosphoproteomics and Signalling Networks

A large-scale integrated study by Wang et al. demonstrated that genetically heterogeneous GBMs frequently converge on shared downstream signalling behaviours mediated by phosphorylation-driven regulatory hubs [96]. In treatment-naïve GBM, integrated proteomic and phosphoproteomic profiling identified phosphorylation of PTPN11 and PLCG1 as key mediators of EGFR-driven signalling, alongside subtype-specific metabolic and immune-associated programs [96]. Extending this work, Liu et al. showed that functional signalling states in high-grade gliomas are more restricted at the protein and post-translational level than at the genomic level [92]. Tumours revealed convergent phosphoproteomic programs centred on signalling integrators such as PTPN11 despite a variety of driver changes. Furthermore, longitudinal analysis showed that tumour recurrence is characterised by extensive proteomic and phosphoproteomic rewiring even when dominant genomic drivers remain stable [92]. All these observations point to a model that suggests adaptive signalling plasticity at the protein and post-translational levels drives the evolution of GBM.

### Translational Insights from Integrated Omics

Growing recognition of GBM plasticity has highlighted the need for improved spatial sampling and longitudinal approaches, such as liquid biopsies, to capture regional heterogeneity, track molecular evolution, and enable adaptive treatment strategies [88].

Omics approaches have revealed therapeutic targets that extend beyond classical genes and pathways [88]. Emerging studies have proposed metabolic and dietary interventions as adjunctive therapeutic strategies. For example, Yuan et al. demonstrated that lysine restriction synergised with MYC inhibition and immune checkpoint blockade to suppress GBM growth, while subsequent work showed that threonine restriction impaired tumour growth in patient-derived xenograft models [97]. These findings, while preliminary, show how metabolic dependencies informed by omics can lead to novel but mechanistically based treatment concepts, albeit dependencies which will require clinical validation to warrant precision medicine potential.

A recurring problem with GBM treatment is that, whereas most research trials do not show a consistent effect, a small percentage of patients frequently respond well. This recurrent trend implies that treatment efficacy reflects underlying molecular heterogeneity that is not captured by traditional patient stratification. This highlights the importance of omics-driven prediction biomarkers that could potentially identify individuals who are most likely to benefit from select treatments [88]. Molecularly informed patient selection, as opposed to tumour-type-based trial design, is likely to represent the future of GBM treatment. Using an integrative multi-omics framework, Saeed et al. identified inflammation-associated genes (IL6, MMP9, and CXCL10) and cell-cycle regulators (CDK1, BUB1, and CCNB2) as network-central hubs whose overexpression correlated with poor patient survival, suggesting these are potential therapeutic targets and prognostic markers in GBM [98].

By allowing the simultaneous analysis of several regulatory layers and the development of composite biomarkers that more accurately reflect tumour state and treatment vulnerability, multi-omics methods have profoundly changed the biomarker discovery process. Through the mapping of cross-layer interactions, integrated frameworks facilitate the development of mechanism-based biomarkers and enable cross-validation of signals across molecular layers, enhancing robustness and repeatability [99]. Additionally, integrative analyses, especially when combined with advanced computational techniques like machine learning, can enhance biologically significant signals that are weak or inconsistent in single-omics analyses [99]. Consequently, multivariate signatures that encode tumour complexity and adaptive behaviour have replaced single molecular features in biomarker discovery [88].

However, there are important methodological issues with this conceptual advancement. Due to overfitting, batch effects, and platform-specific biases, the integration of high-dimensional, heterogeneous datasets significantly raises the probability of false-positive discovery [88]. Disparities in signal-to-noise ratios further distort feature contribution, and differences in data structure across omics layers (continuous transcriptomic measurements versus discrete genomic characteristics) complicate weighting strategies during integration [100].

Despite these drawbacks, emerging applications demonstrate the translational potential of well-designed multi-omics biomarker frameworks. Personalised drug screening based on tumour-specific molecular characteristics has been made possible by integrated omics profiling of patient-derived organoids, creating a useful link between molecular profiling and therapeutic decision-making [101]. Simultaneously, artificial intelligence techniques have demonstrated promise in detecting markers of treatment sensitivity and resistance in GBM as well as in extracting predictive signatures from complicated multi-omics datasets [88]. The interpretability and adaptability of these models, however, continue to be major issues, highlighting the importance of limiting algorithmic complexity and giving biologically transparent frameworks priority.

## Conclusion

The translational impact of integrated omics is most likely to be realised through structured incorporation into clinical workflows and trial design [88], whereby prospective integration enables molecular stratification, longitudinal monitoring, and better therapy selection [102]. Compared to single markers, multi-omics data (coordinated genomic, epigenomic, and proteomic features) are especially useful for defining composite tumour states that may better predict therapy response. However, outputs that are reproducible, interpretable, and actionable within multidisciplinary frameworks are necessary for effective clinical translation [52].

Patient heterogeneity remains the most fundamental challenge, as GBM evolves under therapeutic pressure, with plasticity enabling malignant cells to alter phenotype and reconfigure signalling pathways, thereby compromising initially targetable vulnerabilities.

To better address this in a clinically accurate manner, single cell and spatial omics studies offers representation of GBM biology, revealing adaptive plasticity, and context-dependent vulnerabilities. However, the promise of precision medicine potential in GBM will depend not only upon technological advances alone, but on rigorous validation, clinical integration, and thoughtful appraisal of feasibility. With these constraints addressed, the rapid rate of recent advancements in neuro-oncology makes the clinical integration of these technologies a realistic prospect for precision-based GBM management.

## Key References

- Tang F, Barbacioru C, Wang Y, Nordman E, Lee C, Xu N, et al. mRNA-Seq whole-transcriptome analysis of a single cell. Nature Methods. 2009;6(5):377–82.
  - *(Outstanding importance) Seminal study first reporting a single-cell digital gene expression profiling array*.
- Neftel C. An Integrative Model of Cellular States, Plasticity, and Genetics for Glioblastoma. Cell. 2019;178(4):835–49.e21.
  - *(Outstanding importance) First comprehensive reporting of integrated cell programs, plasticity and genetic driver modulation, revealing four distinct GBM cellular states*.
- Lee Y, Lee M, Shin Y, Kim K, Kim T. Spatial Omics in Clinical Research: A Comprehensive Review of Technologies and Guidelines for Applications. Int J Mol Sci. 2025;26(9).
  - *(Of importance) Recent topical overview of key spatial omics technologies, analytical methods, validation strategies, and clinical applications*.
- Ren Y, Huang Z, Zhou L, Xiao P, Song J, He P, et al. Spatial transcriptomics reveals niche-specific enrichment and vulnerabilities of radial glial stem-like cells in malignant gliomas. Nat Commun. 2023;14(1):1028.
  - *(Outstanding importance) Identification of clinically relevant transcriptional programs, RNA isoform diversity, and multi-cellular ecosystems across different high grade glioma niches*.

## Data Availability

No datasets were generated or analysed during the current study.
